# Effects of lead from ammunition on birds and other wildlife: A review and update

**DOI:** 10.1007/s13280-019-01159-0

**Published:** 2019-03-16

**Authors:** Deborah J. Pain, Rafael Mateo, Rhys E. Green

**Affiliations:** 1 Department of Zoology, University of Cambridge, David Attenborough Building, Pembroke Street, Cambridge, CB2 3QZ UK; 2 Wildfowl & Wetlands Trust, Slimbridge, Gloucestershire GL2 7BT UK; 3 Toxicología de Fauna Silvestre, Instituto de Investigación en Recursos Cinegéticos (IREC), CSIC-UCLM-JCCM, Ronda de Toledo 12, 13005 Ciudad Real, Spain

**Keywords:** Birds, Bullets, Gunshot, Lead, Poisoning, Review

## Abstract

Poisoning of wild birds following ingestion of lead from ammunition has long been recognised and considerable recent research has focused on terrestrial birds, including raptors and scavengers. This paper builds upon previous reviews and finds that both the number of taxa affected and geographical spread of cases has increased. Some lead may also be absorbed from embedded ammunition fragments in injured birds which risk sub-lethal and welfare effects. Some papers suggest inter-specific differences in sensitivity to lead, although it is difficult to disentangle these from other factors that influence effect severity. Sub-lethal effects have been found at lower blood lead concentrations than previously reported, suggesting that previous effect-level ‘thresholds’ should be abandoned or revised. Lead poisoning is estimated to kill a million wildfowl a year in Europe and cause sub-lethal poisoning in another ≥ 3 million. Modelling and correlative studies have supported the potential for population-level effects of lead poisoning in wildfowl, terrestrial birds, raptors and scavengers.

## Introduction

Lead toxicity to humans has been known for centuries, attracting considerable attention as a public health issue in the late twentieth century when longitudinal studies highlighted irreversible effects of low-level chronic exposure to lead on children’s IQ. Introduced legislation subsequently controlled or eliminated many uses of lead (e.g. in petrol and paint) to reduce exposure (Stroud 2015). Lead poisoning of wildlife from ammunition (gunshot or bullets) has been recognised for over a century (Calvert 1876). Birds suffer lead poisoning following direct ingestion (i) of spent lead gunshot from the environment or (ii) of ammunition or ammunition-fragments embedded in their food. (i) is widespread among wildfowl and terrestrial game birds, especially those with a muscular gizzard that eat grit to help grind their food; (ii) among raptors and scavenging birds that eat birds and mammals shot by people, or their discarded remains. An extensive literature links avian lead poisoning to ammunition sources. This includes experimental evidence of dose-dependent effects and field evidence of source, pathway and effects including: ammunition fragments in the alimentary tract of dead and living birds (through post-mortem and x-radiography examinations); ammunition fragments in regurgitated pellets (primarily of raptors); temporal and spatial correlations between elevated tissue lead levels in birds and hunting activities; spatial analyses of elevated tissue lead concentrations in relation to potential sources of exposure to spent ammunition and lead isotopic studies to match tissue lead concentrations with sources. Studies provide overwhelming support for ammunition-derived lead being the major contributor to elevated tissue lead concentrations in wild birds. However, while substantial progress has been made at reducing human exposure to lead from a variety of sources, progress with reducing wildlife exposure to lead from ammunition has been patchy and sometimes ineffective. Lead gunshot has been banned and replaced with non-toxic alternative ammunition types in some places or for some uses (e.g. for all wildfowl hunting in the USA from 1991/92 and all shooting in Denmark from 1996). Many reviews, workshops and conferences have considered this subject in recent decades. Of particular significance are the proceedings of: an International Waterfowl and Wetlands Research Bureau Workshop (Pain 1992); a conference in the USA convened by The Peregrine Fund addressing the implications of lead from spent ammunition for both wildlife and human health (Watson et al. 2009); a symposium held at Oxford University in the UK (Delahay and Spray 2015) and the publication of the final report of the Lead Ammunition Group (LAG 2015), set up to advise the UK government on risks from lead ammunition and mitigation options. A recent proposal for a European Union-wide restriction on the use of lead gunshot for shooting in and over wetlands also includes a detailed evidence review (ECHA 2017). Two scientific consensus reports draw attention to the overwhelming support given by environmental and public health scientists to evidence about the toxic effects on humans and wildlife of lead from ammunition and the need to prevent them (Group of Scientists 2013, 2014). In the following review we do not attempt to repeat previous reviews. Instead, we summarise their key conclusions and update them with results from the substantial literature published during the last 5 years. We evaluate whether the evidence underlying previous conclusions has been reinforced or refuted and highlight areas of research where understanding has been significantly advanced.

## Methods

A literature search using ‘Web of Science’ was conducted for the period 2013–2018 using the following search terms: Lead, ammunition; lead, ammunition, poisoning; bird/reptile/amphibian/mammal/invertebrate/fish and lead and poisoning; bird/reptile/amphibian/mammal/invertebrate/fish and lead and ammunition/bullet/shot; lead, shot, poisoning; lead, bullet, poisoning; lead, game (sorted by animal and poisoning); game; wildlife, lead, poisoning; fishing, lead, poisoning; angling, lead, poisoning; wildfowl, lead, poisoning; waterfowl, lead, poisoning; blood, lead, bird; scavenger, lead, poisoning; vulture, lead, poisoning; plant, lead, ammunition. Preliminary searches of other databases were subsequently undertaken (e.g. Google Scholar and simple ‘Google’ online searches) but it soon became evident that the vast majority of relevant literature had been identified by Web of Science. References that the authors considered added to previous reviews, expanded information or contradicted the conclusions of previous reviews are included below. For brevity, material that simply confirmed the conclusions of previous reviews, or dealt with methodological issues not directly relevant to current knowledge of the effects of lead from ammunition on wildlife, generally has not been included. The literature search was initially conducted in summer 2018 and updated at the end of December 2018.

## Effects of lead on wildlife

Lead is a toxic non-essential metal that has no compensatory beneficial effects in living organisms. It is an accumulative metabolic poison that is non-specific, affecting a wide range of physiological and biochemical systems including the haematopoietic, vascular, nervous, renal, immune and reproductive systems. It causes adverse effects on behaviour and survival (Eisler 1988; ATSDR 2007; EFSA 2010). After absorption by a vertebrate animal, inorganic lead effects are independent of its source. Birds are the most studied and probably the most affected taxon with respect to poisoning from the ingestion of lead from ammunition. However, the toxic effects of lead are broadly similar in all vertebrates and well known from numerous experimental and field studies (reviewed in Eisler 1988; Pattee and Pain 2003; Franson and Pain 2011). Clinical signs of poisoning in birds are often associated with chronic extended exposure at a level that is not initially likely to cause immediate failure of biological function or death, although death may result. Signs include anaemia, lethargy, muscle wastage and loss of fat reserves, green diarrhoea staining the vent, wing droop, loss of balance and coordination and other neurological signs such as leg paralysis or convulsions (e.g. Wobester 1997; Friend and Franson 1999; Pattee and Pain 2003). In contrast, after acute exposure to high levels of lead, birds die rapidly without such signs.

After ingestion of ammunition or ammunition fragments by birds, lead may be eliminated rapidly from the alimentary tract with little lead absorption, retained until completely eroded, solubilised and absorbed, or show any intermediate outcome. Absorbed lead is transported in the bloodstream and deposited rapidly into soft tissues, primarily the liver and kidney, but also into bone and the growing feathers of birds. Lead in bone is retained for long periods and accumulates during an animal’s lifetime, whereas lead in soft tissues has a much shorter half-life (weeks to months). Blood lead (PbB) remains elevated for weeks or months after exposure. Previously suggested ‘thresholds’ to guide the interpretation of tissue lead concentrations are given in Table 1. The physiological effects of lead in birds have been widely reviewed (e.g. Pain and Green 2015). An individual bird’s susceptibility to lead poisoning is influenced by many biological and environmental factors and the sensitivity to lead seems, to some degree, to vary between species. In addition to the direct impacts of lead on welfare and survival, indirect effects are likely to occur. These may include increased susceptibility to infectious diseases, parasite infestations (via lead’s immunosuppressive effects), and increased susceptibility to death from a range of other causes, such as collision with power lines (Kelly and Kelly (2005), Ecke et al. (2017)—via its effects on muscular strength and coordination) and being shot (e.g. shown by Bellrose 1959; reviewed in Pain and Green 2015).

**Table 1:** Suggested interpretation of tissue lead concentrations in Anseriformes, Falconiformes and Accipitriformes

	Blood (µg/dl)	Liver (mg/kg ww)	Kidney (mg/kg ww)	Bone (mg/kg dw)^c^
Sub-clinical poisoning	20 < 50	2 < 6	2 < 6^a^	10–20
			2 < 4^b^	
Clinical poisoning	50–100	6–10	6–15^a^	> 20
			4−6^b^	
Severe clinical poisoning	> 100	> 10	> 15^a^	
			> 6^b^	

Adapted from Franson and Pain (2011) Table 16.1

^a^Anseriformes

^b^Falconiformes and Accipitriformes (previously grouped under Falconiformes in Franson and Pain (2011))

^c^Lead concentrations in bone reflect lifetime accumulation and concentrations may be similar in cases of short-term acute exposure and long-term chronic exposure

It is currently considered that there are no identified “no observed adverse effect levels” (NOAEL) or “predicted no effect concentrations” (PNEC) for lead in humans (EFSA 2010) and the same is thus likely for other vertebrates. Hence, the use of acceptable thresholds for exposure to lead involves acceptance of some level of avoidable harm.

## Pathways of exposure

### Movement of lead derived from spent ammunition into animals via water, soil and plants

The deposition of lead ammunition into the environment can result in elevated soil and water concentrations in relation to the amount deposited and environmental conditions. Wild animals can be exposed to ammunition-derived lead from water or the ingestion of contaminated soil, or via plants or lower organisms that have taken up such lead (reviewed in LAG 2015; Pain and Green 2015). Comparatively little information exists on wildlife effects via these pathways relative to the direct ingestion of lead ammunition or fragments. Recent studies on non-avian taxa support uptake of and effects of ammunition-derived lead via these pathways. One recent study (Rodríguez-Seijo et al. 2017) found high lead levels in soils and the whole bodies of the lumbricid worm *Eisenia andrei* associated with an abandoned shooting range in northwest Spain. High contents of lead and Polycyclic Aromatic Hydrocarbons (PAH) in soil samples and in *E. andrei*, were associated with a reduction in the number of juveniles produced [although this was from both PAH and Pb combined], whereas, *Vibrio fischeri, Raphidocelis subcapitata* and *Daphnia magna* displayed a slight toxic response to the soil leachates tested.

Mariussen et al. (2017) studied the accumulation of lead (Pb) in brown trout (*Salmo trutta*) from Lake Kyrtjønn within an abandoned shooting range in Norway, compared to a nearby reference site (both lakes were acidic). Brown trout from Lake Kyrtjønn had significantly elevated lead in bone and other tissues and significantly inhibited ALAD activity in the blood compared to those from the reference site. Trout eggs were placed in stream outlets from both lakes and lead concentrations were significantly elevated in eggs and alevins from Lake Kyrtjønn compare to the reference lake. The authors concluded that adult brown trout, fertilised eggs and alevins, may be subjected to increased stress due to chronic exposure to Pb.

### Direct ingestion of spent lead ammunition from the environment

Lead gunshot has been used for centuries. An estimated 600–700 million cartridges containing lead gunshot (18 000–21 000 tonnes of lead; c. 200 thousand million individual gunshot) are used annually in Europe for hunting. Some of it instantly kills the animals at which it is fired, but a proportion of them are wounded. The viscera of killed animals of some species, such as deer, are discarded in the environment. Only a small proportion of gunshot strike their targets, and so the remaining spent ammunition is dispersed widely into the environment (ECHA 2017). Under most environmental conditions, metallic lead is relatively stable. Over time, ammunition lead deposited on the soil or in water will degrade through a process of erosion and chemical reaction and lead compounds with different solubilities may form on its surface. Soil lead concentrations generally increase, especially in areas of high deposition and/or when soil conditions, such as low pH, facilitate this. Degradation of lead shot is slow, probably taking tens to hundreds of years. Thus a “historical legacy” of gunshot remains available to wildlife, increasing over time where shooting with lead gunshot continues. Gunshot generally sinks slowly through most types of soil and mud and may be available to feeding birds for many years, although a high proportion of gunshot ingested is that most recently deposited. Pain et al. (2015) review this in relation to soil types and management practices.

**Fig. 1:**
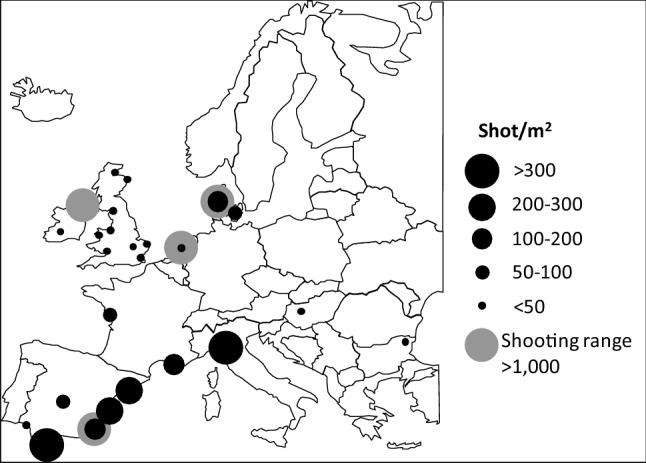
Densities of lead shot in wetlands due to waterfowl hunting and sport shooting. Modified from Descalzo and Mateo (2018), updated from Mateo (2009). Shot densities are from individual or multiple sites in each country and from depths ranging from 5 to 30 cm. Data from Table 2 of Descalzo and Mateo (2018), updated from Mateo (2009)

Gunshot densities are higher in areas of intense or regular shooting. In wetlands, densities may range from just a few to several hundred gunshot/m^2^ (Fig. 1) but thousands/m^2^ can be found in some situations like target shooting areas (e.g. O’Halloran et al. 1988).

**Fig. 2:**
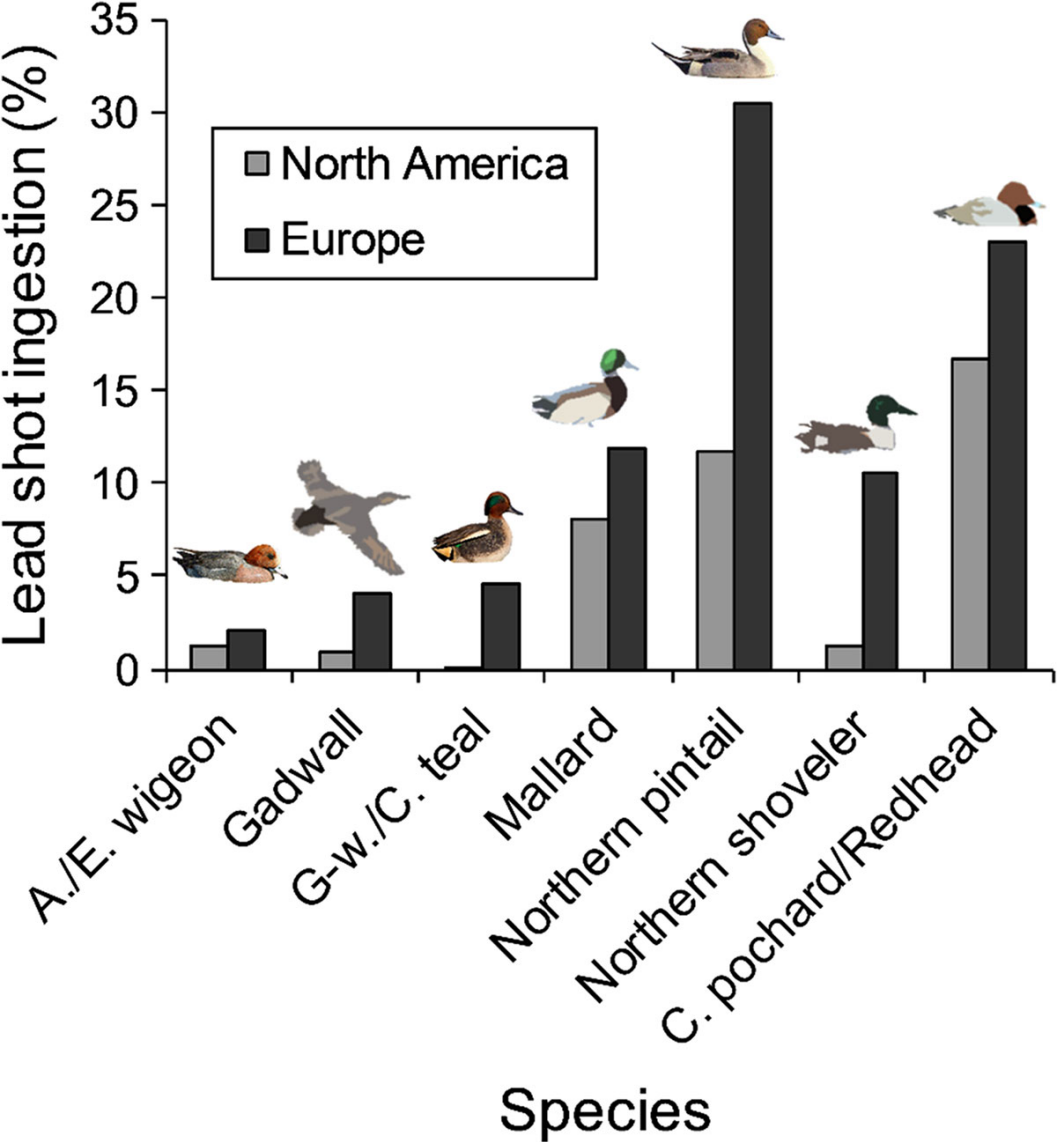
Prevalence of Pb shot ingestion in waterfowl species from North America (*n* = 171 697) and Europe (*n* = 75 761). Modified from Mateo (2009). American wigeon (*Anas americana*), Eurasian wigeon (*Anas penelope*), gadwall (*Anas strepera*), green-winged teal (*Anas carolinensis*), common teal (*Anas crecca*), mallard (*Anas platyrhynchos*), northern pintail (*Anas acuta*), northern shoveler (*Anas clypeata*), common pochard (*Aythya ferina*), redhead (*Aythya americana*)

There is evidence of direct ingestion of spent ammunition from soil or mud by many species of birds including wildfowl, some other water birds and game birds across Europe, North America and other countries where studies have been conducted. A substantial body of evidence spanning more than half a century exists documenting this pathway (e.g. Mateo 2009; Pain and Green 2015). Reported levels of gunshot ingestion, in terms of the proportions of killed or trapped birds with shot in the alimentary tract vary among wildfowl species according to their feeding habits. Species that feed on seeds and hard-bodied benthic animals, such as molluscs, tend to ingest larger particles of grit to assist in breaking up their food, whereas species that graze plant leaves ingest small grit particles. For a given wildfowl species, or for species with comparable feeding ecology, the proportion of birds with ingested shot tends to be higher in Europe than North America (Fig. 2). Few recent papers add to the literature on this pathway, presumably because the literature is already vast and few questions remain unanswered. Also, legislation has been introduced in some places to limit exposure—although this is incomplete and has met with variable success (see papers is Delahay and Spray 2015). The few recent studies on this pathway support previous conclusions of exposure where a pathway exists, and further document the wide geographical extent of the problem and range of species affected.

Recent European studies have reported little or no evidence of gunshot ingestion in geese feeding in areas with no hunting or low gunshot densities (Aloupi et al. 2015; Mateo et al. 2016 for studies in Greece and Bulgaria respectively). Runia and Solem (2016) found levels of lead gunshot ingestion to be about five times higher in 660 ring-necked pheasant (*Phasianus colchicus*) harvested on shooting preserves in South Dakota USA then in 1301 birds from non-preserve areas where lead gunshot availability was presumably lower (3.9%, 95% CI 2.7–5.7% vs 0.8%, 95% CI 0.4–1.4% respectively).

Mateo et al. (2013) reviewed lead poisoning studies from Spain and reported that high densities of lead gunshot in various internationally important wetlands for waterfowl resulted in proportions of birds with ingested gunshot close to 70% in some species, such as pintail (*Anas acuta*). This study found that lead poisoning is a major cause of mortality of the white-headed duck (*Oxyura leucocephala*), listed as Endangered in the IUCN Red List. High proportions of birds with ingested gunshot (9.3% of 461 birds) were reported in chukars (*Alectoris chukar*) harvested in north-western Utah, USA, and 8.3% of 121 birds analysed had elevated liver lead (Bingham et al. 2015). Few studies have previously been published from Argentina. Ferreyra et al. (2014) investigated gunshot ingestion and blood lead concentrations in 415 hunter-killed ducks and 96 live-trapped ducks of 5 species in Argentina. Overall 10.4% of ducks contained ingested gunshot. Blood lead levels were elevated in 28% of ducks and exceeded 100 µg/dl, a threshold for clinical toxicity, in 8.6% of birds. Lead poisoning has been reported in the globally Vulnerable nene (*Branta sandvicensis*) in Hawaii (Work et al. 2015). Recent studies from Iranian wetlands found elevated levels of lead in the livers and/or kidneys of some sampled common pochard (*Aythya ferina*), mallard (*Anas platyrhynchos*), teal (*Anas crecca*) and gadwall (*Anas strepera*), with ingested gunshot suggested as a possible source (Sinkakarimi et al. 2015, 2018).

Using lead isotope analyses of blood lead levels, Binkowski et al. (2016) concluded that lead gunshot currently available in Poland was unlikely to be the source of blood lead (mean of 0.241 ppm—24 µg/dl) in mute swans (*Cygnus olor*) wintering in northern Poland, although these authors did not examine how the relationship between blood and ammunition isotope ratios changes with blood lead concentration, and ^204^Pb, upon which this conclusion relies, is not readily analysed using Q-ICP-MS (Ellam 2010).

### Ingestion of lead from ammunition by raptors and scavengers

Lead from ammunition is available to predators and scavengers in the flesh of their prey either as whole gunshot/bullets or ammunition fragments. Exposure occurs when shot animals are killed but not retrieved, when parts of the carcass (e.g. offal) are discarded, or when animals are wounded but survive (and may be more vulnerable to early death later or to predation). Large numbers of some quarry species can survive carrying lead gunshot, commonly 20–30% in some wildfowl populations (Table I, Pain et al. 2015). Recent studies investigating the presence of lead fragments and/or elevated tissue lead concentrations add to evidence of the contamination of both small and large game species with lead ammunition (Warner et al. 2014; Cruz-Martinez et al. 2015; Andreotti et al. 2016; Ertl et al. 2016; Herring et al. 2016). Using wild and captive birds (ravens (*Corvus corax*), white-tailed eagles (*Haliaeetus albicilla*) and common buzzards (*Buteo buteo)*) Nadjafzadeh et al. (2015) found that birds more frequently ingested smaller metal fragments. However, they only avoided those > 8.8 mm, which is considerably larger than most gunshot or bullet fragments. Analyses of ‘trash’ items from the nest area of California condors (*Gymnogyps californianus*) (Finkelstein et al. 2015) and a literature review (Golden et al. 2016) confirmed the view that, while different sources of lead are available in the landscape, most lead poisoning of scavenging birds appears to result from lead-based ammunition ingested in their food. Low tissue lead levels (liver lead < 2.1 ppm dw; *N* = 11) have been reported in the lesser-spotted eagle (*Clanga pomarina brehm*), a species that breeds in Europe but migrates to Africa, thus avoiding the European hunting season (Kitowski et al. 2017c). In contrast, a greater-spotted eagle (*Clanga clanga*), a related species that is associated with wetlands and susceptible to lead poisoning as it feeds on unretrieved quarry (BirdLife International 2018), was found wintering in the Ebro Delta, Spain, with an elevated blood lead level of 33.6 µg/dl (Mateo et al. 2001).

Previously, most studies documenting this exposure pathway were of condors and eagles (particularly California condor, bald eagle (*Haliaeetus leucocephalus*) and golden eagle (*Aquila chrysaetos*) in North America, white-tailed eagle in Europe and Japan and Steller’s sea eagle (*Haliaeetus pelagicus*) in Japan). However, many other species have been reported as exposed to and/or poisoned by lead ammunition. While fewer studies had been conducted on other species, recent research has documented exposure via this pathway in new species, taxa and locations, considerably strengthening the evidence-base and highlighting the significance of this exposure pathway. These studies are summarised in Table 2, to which previous studies, reviewed in Pain et al. (2009) add at least another 14 species.

**Table 2:** Recent studies on exposure to and poisoning from lead from ammunition in predatory and scavenging birds

Species	Location	Evidence^d^	References
Andean condor^a,b^*Vultur gryphus*	Argentina	Wild condors from rehabilitation centres (*n* = 62) had high lead levels (mean PbB 15.47 µg/dl, max. 104 µg/dl; bone lead* mean 22 ppm, max 148.20 ppm) compared with captive bred individuals (mean PbB 5.63 µg/dl, and mean bone lead* 2.76 ppm, *n* = 10). Two wild birds had lead in the gastrointestinal tract and 13 had lead fragments elsewhere in their bodies * sampled from live birds	Wiemeyer et al. (2017)
	Chile	Two birds reported with high PbB (one bird—89 µg/dl) and liver and kidney levels (one bird—136 and 247 ppm respectively) after ingesting lead bullets	
Bearded vulture *Gypaetus barbatus*	Swiss Alps	Two of five birds found dead had bone lead concentrations consistent with lead poisoning (59 and 100 mg/kg)	Ganz et al. (2018)
White-backed vultures^a^ *Gyps africanus*	South Africa and Namibia	PbB compared in wild and captive birds. 12% of wild birds appeared to be exposed to an additional source of lead than purely environmental—presumed to be ammunition	Naidoo et al. (2017)
	Botswana	PbB was analysed from 566 wild captured birds. 30.2% had elevated PbB (10 to 45 μg/dl) and 2.3% had PbB ≥ 45 μg/dl. PbB levels were higher in samples taken during than outside the hunting season and from within hunting rather than outside hunting areas	Garbett et al. (2018)
Griffon vulture *Gyps fulvus*	Israel	A sick bird found with a 9 mm lead bullet in the proventriculus and a PbB of 805 µg/dl subsequently died of lead poisoning	Horowitz. et al. (2014)
	French Pyrenees	In a surveillance programme for avian scavenger populations 3 of 119 birds reported as dying of lead poisoning. Isotopic signature consistent with ammunition source	Berny et al. (2015)
	Aragón, NE Spain	691 blood samples were collected over 5 years and analysed for PbB and Pb isotopes with statistical modelling used to investigate sources and spatio-temporal distribution of PbB. While isotope signatures overlapped considerably, naturally occurring sources of lead were considered to result in a high proportion of birds having moderately elevated PbB (45% of birds had > 20 µg/dl), with point sources (e.g. lead-based ammunition) associated with high PbB, as illustrated by different isotope ratios between birds with PbB < 20 µg/dl and > 47 µg/dl	Mateo-Tomás et al. (2016)
	Iberian peninsula	Three birds were found sick treated in a wildlife rehabilitation centre but died with PbB 969–1384 µg/dl, liver Pb 309–1077 µg/g dw, kidneys Pb 36-100 µg/g dw. Nine un-eroded lead pellets recovered from the stomach of one bird	Carneiro et al. (2016)
Cape vulture^a^*Gyps coprotheres*	South Africa and Namibia	PbB compared in wild and captive birds. 31% of wild birds appeared to be exposed to an additional source of lead than purely environmental—presumed to be ammunition. One bird with a PbB of 100 µg/dl died soon after capture	Naidoo et al. (2017)
Golden eagle *Aquila chrysaetos*	Swiss alps	Tissues of 41 dead, injured or moribund eagles were examined. Pb distributions in blood and soft tissues were right skewed. In 22% of birds only one of the three flight feather segments had a high Pb concentration. Results suggest episodic and repeated lead intake likely resulting from ammunition in offal or carcasses	Jenni et al. (2015)
	Swiss alps	Three birds were found with acute lead poisoning (56 and 108 µg/dl PbB and 77 ppm d.w. liver Pb). In a comparative study (31 eagles and 19 Eagle Owls *Bubo bubo*) the authors found that lead isotope signatures of Golden Eagle bones were similar to those of ammunition, but differed from the signatures of bones of their prey, Eagle Owls (that do not scavenge) and soil	Madry et al. (2015)
	Swiss Alps	Liver and/or bone Pb concentrations were analysed in 67 dead golden eagles; 31 were previously published (Madry et al. 2015; Jenni et al. 2015) as shown above in this table and 36 were from new birds. Five of 55 birds had liver Pb > 6 mg/kg, the highest being 77 and 80 mg/kg. Fourteen of 46 birds had bone Pb > 20 mg/kg. These lead burdens were higher than those found elsewhere in Europe or North America and compatible with acute lead poisoning	Ganz et al. (2018)
	North America	10% of 178 golden eagles captured during fall migration were clinically lead poisoned (PbB > 0.6 mg/l [60 µg/dl]) and 4% were lethally exposed (PbB > 1.2 mg/l [120 µg/dl]). PbB was higher in golden eagles captured on carrion than those captured using live bait	Langner et al. (2015)
	Northern Sweden	PbB in wild birds was significantly correlated with the progress of the moose hunting season. One bird starved with PbB of 57.3 µg/dl increasing from 26.7 µg/dl after trapping and one bird collided with a powerline with a PbB of 38.9 µg/dl. Deaths may be associated with lead poisoning directly or indirectly	Ecke et al. 2017
	USA	4.7% of 1427 deaths between 1975 and 2013 from lead poisoning	Russell and Franson (2014)
Bonelli’s eagle^a^ *Aquila fasciata*	Granada, south-eastern Spain	Lead shot in 2.81% of 1 363 regurgitated pellets in spring and 1.32% of 172 pellets in autumn (from a total of 14 territories) coinciding with hunting seasons of prey. Shot were found in pellets in 8 of the 10 territories for which 20 or more pellets were collected. The frequency of occurrence of shot was positively related to feather lead concentrations.	Gil-Sánchez et al. (2018)
Red kite *Milvus milvus*	England	Lead poisoning was diagnosed in 6 of 110 red kites found dead between 1989 and 2007	Molenaar et al. (2017)
	French Pyrenees	Lead poisoning was reported as cause of death of 4 of 34 birds found dead in a surveillance programme for avian scavenger populations. Lead isotope ratios were consistent with an ammunition source	Berny et al. (2015)
White-tailed eagle *Haliaeetus albicilla*	Hokkaido, Japan	12 of 50 dead birds collected after a ban on the use of lead bullets for hunting sika deer (*Cervus nippon yesoensis*) had elevated liver lead concentrations (> 2 ppm w.w.; max. 56.4 ppm) associated with poisoning. Isotope analysis was consistent with lead ammunition	Ishii et al. (2017)
	Poland	7 of 22 birds found dead or moribund had liver lead levels > 30 ppm dw, two of which were 180.3 and 188.6 ppm dw. This was apparently associated with feeding on wintering waterfowl and carrion	Kitowski et al. (2017a)
	Finland	Lead poisoning was the most important cause of human-related mortality in 123 carcasses of white-tailed eagles (collected 2000–2014) accounting for 31% of all cases	Isomursu et al. (2018)
	Ireland	A white-tailed eagle at Lough Derg died of lead poisoning believed to be associated with the bird feeding on wildfowl that had been shot with lead shot	O’Donoghue (2017)
Bald eagle *Haliaeetus leucocephalus*	USA—Upper Mississippi River Valley	PbB was higher immediately following the hunting season and lower when the previous months’ snowfall was greater than 11 cm, when game animal carcasses may be concealed	Lindblom et al. (2017)
		16.3% of 2980 bald eagle deaths between 1975 and 2013 were from lead poisoning. The proportion of lead-poisoned eagles increased in all 4 migratory bird flyways of the United States after the autumn 1991 ban on the use of lead shot for waterfowl hunting, probably as Bald Eagles consume lead ammunition fragments (from bullets) in offal and carcasses left behind during big game hunting seasons	Russell and Franson (2014)
	USA—Iowa	31% of 209 eagles brought to rehabilitation centres (2004-2014) had PbB > 60 µg/dl. 30 of 59 birds for which livers were analysed post-mortem had liver Pb > 6.0 ppm ww, suggestive of clinical poisoning	Yaw et al. (2017)
	USA—Iowa, Minnesota and Wisconsin Canada—Ontario	38% of 58 birds found had liver Pb > 6 ppm ww. Birds were from areas where 36% of discarded offal piles from hunter-killed deer were found to contain lead fragments.	Warner et al. (2014)
		23% (10/43) of birds found dead and dying between 1991 and 2008 died of lead poisoning	Martin et al. (2018)
Steller’s sea eagle *Haliaeetus pelagicus*	Hokkaido, Japan	18 of 43 dead birds collected after a ban on the use of lead bullets for hunting sika deer had elevated liver lead concentrations (> 2 ppm w.w.) associated with poisoning. Isotopic analysis was consistent with lead ammunition. One bird that died in 2013 had a lead bullet in the stomach and a liver lead of 36.3 ppm ww	Ishii et al. (2017)
Common buzzard *Buteo buteo*	Portugal	Authors reported an apparent association between PbB and the hunting season	Carneiro et al. (2014)
	Poland	Foraging on carrion and game carcasses was associated with elevated (> 6 ppm dw) liver Pb concentrations in some buzzards; 3 of 31 birds taken to rehabilitation centres that later died had liver Pb > 6 ppm dw with one bird exceeding 15 ppm dw	Kitowski et al. (2016)
Southern ground hornbill^a^*Bucorvus leadbeateri*		Authors reported acute lead poisoning in a bird. Lead particles in the gizzard were probably from the carcass of a porcupine (*Erethizon dorsatum*) that was killed with lead shot. Two other birds reported as likely to have been exposed	Koeppel and Kemp (2015)
Peregrine falcon *Falco peregrinus*	Italy	Dead adult female (cause of death unknown) had many lead shot in the digestive tract, mixed with the remains of a feral pigeon (*Columba livia domestica*) and a European starling (*Sturnus vulgaris*)	Andreotti et al. (2018b)
Common raven *Corvus corax*	Eastern Quebec, Canada	PbB in birds captured during two moose hunting seasons increased as the hunting seasons progressed, with over half of birds having PbB > 10 µg/dl during the hunting season and the Pb isotope signature in contaminated ravens tended towards the lead ammunition signature	Legagneux et al. (2014)
	California (northern)	Birds captured during the hunting season (*n* = 10) had median blood lead levels almost sixfold higher than those captured during the non-hunting season (*n* = 17)	West et al. (2017)
Rook *Corvus frugilegus*^a^ Hooded Crow *Corvus Cornix* ^a^ Magpie *Pica pica*^a^	Eastern Poland	Liver lead concentrations were measured in birds taken to rehabilitation centres that subsequently died; an individual rook (6.33 ppm dw, *N* = 1 of 24), hooded crow (21.77 ppm dw, *N* = 1 of 6) and magpie (8.62 ppm dw, *N* = 1 of 2) had liver lead levels > 6ppm dw. The authors considered that elevated levels may result from these species’ propensity to scavenge on animals killed with lead ammunition or eat grit/small stones and mistakenly ingest spent ammunition	Kitowski et al. (2017b)
Studies of lead contamination with no source identified			
Griffon vulture *Gyps fulvus*	Iberian peninsula	22% (44 of 54) wild caught birds had PbB > 20 µg/dl and one of these had PbB > 100 µg/dl. The origin was not determined but the authors speculated that lead from rubbish dumps or ammunition in carcasses were possibilities	Carneiro et al. (2015)
American black vulture^a^*Coragyps atratus* and turkey vulture *Cathartes aura*	Virginia, USA	Wild black (*n* = 98) and turkey vultures (*n* = 10) were culled in Virginia, USA and tissues analysed for Pb and Pb isotopes. Mean bone levels in all birds indicated chronic exposure to lead (37 ppm in black and 23 ppm in turkey vultures) but few birds had elevated liver lead indicative of recent exposure (mean of 0.78 ppm for black and 0.55 ppm turkey vultures). Tissue isotopic ratios overlapped a range of potential contributing sources including ammunition, gasoline, coal-fired power plants and zinc smelting	Behmke et al. (2015)

			References
Predatory and scavenging bird species from previous studies of exposure to and poisoning from lead from ammunition^c^			
White-rumped vulture (*Gyps bengalensis*), Egyptian vulture (*Neophron percnopterus*), cinereous vulture (*Aegypius monachus*), Eastern marsh harrier (*Circus spilonotus*), Western marsh harrier (*Circus aeruginosus*), Northern harrier (*Circus cyaneus*), Eurasian sparrowhawk (*Accipiter nisus*), sharp-shinned hawk (*Accipiter striatus*), Cooper’s hawk (*Accipiter cooperii*), Northern goshawk (*Accipiter gentilis*), red-tailed hawk (*Buteo jamaicensis*), rough-legged buzzard (*Buteo lagopus*), Spanish imperial eagle (*Aquila adalberti*), American kestrel (*Falco sparverius*) plus several additional species of raptor in captivity, several species of owl (captive and wild) and several species of gull (wild)			Reviewed in Pain et al. (2009)

^a^We have no knowledge of previous reports in the published literature of lead poisoning in the wild in this species; for Andean condor, elevated feather lead with isotopic signals compatible with ammunition sources had been reported previously from Patagonia in Argentina (Lambertucci et al. 2011)

^b^Ingestion of or poisoning by lead from ammunition has been reported in captive birds—see review of Pain et al. (2009)

^c^Excluding species already listed above in the table

^d^Concentrations are given in the units presented in the references: ppm = µg/g = mg/kg

New studies highlight the potential for exposure of mammalian scavengers and predators to ammunition lead. Legagneux et al. (2014) used camera traps to identify species scavenging on moose (*Alces alces*) viscera left by hunters in eastern Quebec, Canada and these included black bears (*Ursus americanus*). In a study of brain metal concentrations in 9 mammal species from north-western Poland, Kalisinska et al. (2016) found that raccoon dogs (*Nyctereutes procyonoides*) from an area where hunting is prohibited had a lower brain lead compared to those from hunting grounds, and speculated that the elevated levels could have resulted from ingesting lead from animals shot by hunters. Two captive cheetahs that had routinely been fed on hunted antelope or game birds were suspected to have died from lead poisoning; they had ingested bullets in their stomachs, elevated tissue lead levels and associated clinical sign of poisoning (North et al. 2015). Hivert et al. (2018) found significantly higher blood lead concentrations in captive than wild Tasmanian devils (*Sarcophilus harrisii*). Captive Tasmanian devils were fed the meat of wild animals shot with lead ammunition. Subsequent removal from the Tasmanian devil’s diet of the lead-containing heads and wounds from shot animals resulted in a significant decrease in blood lead concentrations in animals at one of the captive study sites. These studies suggest that mammalian predators and scavengers that eat game species may also be at risk.

### Movement of lead from embedded ammunition into body tissues

It is well established that lead from ammunition that has been shot into and become embedded in human body tissues can be mobilised and give rise to health effects (Weiss et al. 2017). Until recently, little was known about this possible pathway of exposure in wildlife. One study found that 2 white-tailed deer (*Odocoileus virginianus)* with retained lead ammunition from previous gunshot wounds had muscle tissue lead concentrations similar to controls although one had elevated bone lead levels (Zimmer and Osier 2018). Other recent work on birds suggests that some embedded lead may be mobilised. Berny et al. (2017) found that birds of prey in French wildlife centres that had embedded lead projectiles had significantly higher blood lead concentrations than those without (22.4 vs 14.3 µg/dl), suggesting that embedded lead projectiles may release lead and have long-term health effects. In Peru, 6 of 9 Andean condors (*Vultur grypphus*) at a rehabilitation centre had detectable blood lead levels (3.7 µg/dl to 17.4 µg/dl), with a mean of 9.95 µg/dl. The highest value was from a condor admitted due to a gunshot wound and found on radiographic examination to be carrying 45 lead pellets embedded in body tissues as confirmed by X-ray examination (L. Schaefer pers. com. cited in Wiemeyer et al. 2017). In a study of wild California condors, Finkelstein et al. (2014) found one bird that had been shot and retained embedded birdshot (small sized gunshot) in its tissues. The blood lead level was 16.6 µg/dl and the isotope ratios of birdshot and blood lead were indistinguishable. The following year the same bird was captured with clinical lead poisoning (blood lead levels 556 µg/dl) and radiography showed that it had ingested a buckshot (large sized gunshot), and still retained embedded birdshot from the previous shooting incident. The blood and buckshot lead isotope ratios were indistinguishable at this time, but the buckshot isotope ratio was measurably different from that of the birdshot. This illustrates that ingested shot presents a far greater risk to this species than embedded shot, but nonetheless that some transference of lead from embedded shot appears to occur. Similarly LaDouceur et al. (2015) measured tissue lead concentrations in 14 individual wildlife cases with embedded lead projectiles that were unrelated to the cause of death. Clinically significant liver lead concentrations were only found in two cases suggesting that embedded lead carries a relatively low risk for lead poisoning.

While the number of studies on this pathway remains small, collectively, they suggest that some of the lead from embedded gunshot may be mobilised, resulting in increased blood lead concentrations, with potential long-term effects. It currently appears that absorption of lead from embedded ammunition is likely to be modest compared with that following the ingestion of lead from ammunition, and that effects may be sub-lethal. Several authors have previously reported associations between embedded gunshot and reduced body condition or survival in birds (Madsen and Noer 1996; Tavecchia et al. 2001; Merkel et al. 2006), but it is unclear whether these effects were related to the injuries caused by shooting, sub-lethal effects of lead absorbed from embedded gunshot, a combination of these or some other explanation.

While effects from this pathway appear likely to be small in comparison to those from ingested lead, large numbers of birds could be affected because a substantial proportion of some populations of game birds survive being shot, carrying gunshot in their flesh (e.g. commonly 20–30% in some wildfowl populations—Pain et al. 2015). Should this pathway be further verified, then numbers of birds suffering welfare effects from sub-lethal poisoning would be far greater than previously supposed.

## Impacts of lead poisoning on wildlife

### Sub-lethal and welfare effects

Most birds that ingest lead ammunition suffer some effects as a result of absorbing above background levels of lead. These may be sub-clinical or clinical and will affect the birds’ welfare to varying degrees. In recent years considerable effort has been put into investigating the sub-lethal effects of lead from ammunition on birds, both under experimental conditions and in the wild.

Using transmission electron microscopy, Pineau et al. (2017) found marked subcellular toxicity in the liver associated with the ingestion of a single lead shot (0.177 ± 0.03 g) in experimentally dosed mallards (*n* = 21) compared with controls (*n* = 10). Experimental studies involving the dosing of pheasants (Gasparik et al. 2012; Runia and Solem 2017) and northern bobwhites (*Colinus virginianus*) (Tannenbaum 2012) with lead gunshot suggest that these species appear less susceptible to the acute effects of lead poisoning than others, such as mourning doves (*Zenaida macroura*), chukars or waterfowl. While tissue (blood and/or liver) lead concentrations of pheasants and northern bobwhites increased, and some of the biological parameters measured were negatively affected (two studies), this was to a lesser degree than in other species. Other experimental work has shown the sensitivity of certain terrestrial species to sub-lethal effects of lead, sometimes at low exposure levels. Maternal consumption of one 95-mg lead pellet affected egg size and hatchling organ development in domesticated roller pigeons (*Columba livia*) (Williams et al. 2017). Vallverdú-Coll et al. (2015a) reported impacts on immune response and other variables in red-legged partridges (*Alectoris rufa*) dosed with 1–3 lead gunshot.

Vallverdú-Coll et al. (2016a) found that female red-legged partridges dosed with three lead pellets (330 mg) had reduced egg hatching rate and males had decreased acrosome integrity and sperm motility. In contrast, when exposed to the lower dose of 1 pellet (110 mg), females produced heavier eggs and chicks and males presented increased sperm vigour. Then authors suggested that at the low exposure levels lead-induced endocrine disruption could explain the production of heavier and larger eggs by exposed females, although this effect has rarely been studied in female birds. Espín et al. (2014) investigated blood lead concentrations that cause effects on oxidative stress biomarkers using blood taken from 66 griffon vultures (*Gyps fulvus*) in Spain, and found that levels > 15 µg/dl can result in oxidative stress, risking damage to cell components. These and previous experimental studies suggest inter-specific variation in susceptibility to lead poisoning. Inter- and intraspecific variation in lead toxicity relates to factors that influence absorption, retention, detoxification and elimination of lead. Among these are diet (considered a key variable influencing lead absorption), age, sex, physiological condition and environmental factors such as temperature (Pattee and Pain 2003). Disentangling intrinsic variation in susceptibility from the effects of experimental conditions is complex.

Several recent studies have reported sub-lethal effects of lead in wild birds, supplementing earlier research. A histopathological study of the eyes of a bald eagle provided the first evidence of ocular lesions associated with sub-lethal but extremely elevated blood lead levels (c. 610 µg/dl—Eid et al. 2016). The prognosis for this rehabilitated bird’s vision was too poor for it to be released back to the wild. Along with other effects, body condition was negatively associated with liver lead of hunter-killed wild ducks in Argentina (Ferreyra et al. 2015) and with blood lead levels of female common eider (*Somateria mollissima*) in Canada (Provencher et al. 2016). Vallverdú-Coll et al. (2016b) found that in mallards from the Ebro delta (north-eastern Spain), lead exposure was associated with increased oxidative stress, affected colour expression, and impaired constitutive immunity in ways that differed between the sexes. Through analysing mineral chemistry and crystallinity, Álvarez-Lloret et al. (2014) found that lead contamination altered bone remodelling of red-legged partridges from a farmland area in Albacete, Spain, in a concentration-dependent way and that this occurred at low bone lead concentrations (< 4 ppm dw bone lead).

Several papers have reported sub-lethal effects at lower blood lead levels (PbB) than previously suggested (Table 1). This mirrors research into the effects of chronic low-level exposure in humans, with reference values for elevated PbB considered significant by the Centers for Disease Control and Prevention decreasing markedly over time (CDC https://www.cdc.gov/). Vallverdú-Coll et al. (2015b) found that lead gunshot ingestion in mallards can result in maternal transfer of lead to offspring, affect their developing immune system and reduce early life stage survival. In mallard eggs from the Ebro delta (Spain) eggshell lead and duckling blood lead levels were positively correlated, and ducklings with blood levels > 18 µg/dl had reduced body mass and died during the first week post hatching. Newth et al. (2016) found elevated (> 20 µg/dl lead in blood) levels in 41.7% (125/300) of whooper swans (*Cygnus cygnus*). Blood lead content was significantly negatively associated with winter body condition when levels were ≥ 44 µg/dl (27/260 = 10.4%), indicating that sub-lethal impacts of lead on body condition occur at the lower end of previously recommended clinical thresholds and that a relatively high proportion of individuals in this population may be affected. Analysis of tracking data from 16 adult golden eagles trapped in northern Sweden indicated that sub-lethal blood lead concentrations reduced mean flight height and movement rate (Ecke et al. 2017). Blood lead levels of c.2.5 µg/dl appeared to reduce flight height by 10%, levels of 4.3 µg/dl by 20%, and in birds with the highest blood levels by 50%. These lead blood concentrations fall far below previously suggested thresholds (Table 1), but because of the small sample the authors suggest that further data should be collected. González et al. (2017) studied blood parameters and lead concentrations in griffon vultures submitted to Wildlife Rehabilitation Centres in Spain. 26% of birds had blood lead > 20 µg/dl. Blood lead was negatively correlated with haematocrit and digestive signs such as stasis and weight loss, though not with other clinical signs. The authors suggested that this species may be more sensitive to the toxic effects of lead than previously thought.

### Deaths from lead poisoning

Few recent studies have added to the substantial body of information on deaths from lead poisoning in wildfowl with most new research on mortality covering the previously less well-studied groups of raptors and scavengers. Despite this disparity, a number of species of raptor and scavenger had previously been reported as dying of lead poisoning in Europe and North America (reviewed in Mateo 2009; Pain and Green 2015; Golden et al. 2016). As awareness of this poisoning in raptors has increased, so too has the number of research studies. Table 2 includes many recent examples of mortality from ingesting lead from ammunition (or likely from ammunition) in raptors and scavengers including Andean condor, cape vulture (*Gyps coprotheres*), griffon vulture, golden eagle, red kite (*Milvus milvus*), white-tailed eagle, bald eagle and Steller’s sea eagle and ranging geographically from Europe, the Middle East and Japan to Africa, North and South America. Many other species had already been reported as being poisoned in this way (e.g. see Pain et al. 2009).

Recent studies illustrate that some partial bans on the use of lead ammunition do little to reduce lead poisoning mortality in raptors and scavengers. In North America, lead poisoning continues to be a significant cause of mortality in bald and golden eagles, despite the ban in autumn 1991 on the use of lead ammunition for shooting waterfowl (Russell and Franson 2014; Warner et al. 2014; Yaw et al. 2017). This is largely because eagles consume lead bullet fragments in offal and carcasses left behind during big game hunting. In Japan, lead poisoning remains a problem for Steller’s sea eagles and white-tailed eagles after partial bans on the use of lead ammunition (first for shooting sika deer (*Cervus nippon*) and then all large game in Hokkaido in 2000, 2001 and 2004—Ishii et al. 2017). Bans on only certain types of lead ammunition (either gunshot or bullets) or only for the shooting of certain animals frequently have limited effect, and can be difficult to police (e.g. see Cromie et al. 2015 for UK example), although at one site in Spain good enforcement improved compliance (Mateo et al. 2014). In California, lead poisoning has been a major factor in both causing the extinction in the wild and then limiting the recovery of the reintroduced California condor population. After successive types of limited ban, a total ban—on the use of all lead ammunition for all hunting—will come into force in California from 2019 (Rendon Act 2013).

### Numbers of birds affected

Sufficient data are available for approximate estimates to be made of annual numbers of deaths caused by lead poisoning in wildfowl. These are necessarily imprecise as many external factors affect lead-poisoning mortality from year to year, notably food availability and weather (Pattee and Pain 2003). Estimates follow the method of Bellrose (1959) and are based on proportions of birds found to have ingested different numbers of gunshot, turnover rates of gunshot in the intestine and mortality from experimental studies. Andreotti et al. (2018a) estimated that about 700 000 individuals of 16 waterbird species die annually in the European Union (EU) (6.1% of the wintering population) and one million across Europe (7.0%) as a direct effect of lead poisoning, with three times more birds suffering sub-lethal effects. This is similar to the number previously estimated by Mateo (2009). Pain et al. (2015) estimated that in the UK in the order of 50 000–100 000 wildfowl (c. 1.5–3.0% of the wintering population) die each winter (i.e. during the shooting season) as a direct result of lead poisoning (Pain et al. 2015), with several hundred thousand birds suffering sub-lethal poisoning and welfare effects. Wildfowl that die from delayed effects outside of the shooting season will be additional, as will those that ingest gunshot outside of the shooting season or die of causes exacerbated by lead poisoning (e.g. infectious diseases, collisions).

Pain et al. (2015) considered estimates of mortality for terrestrial game birds in the UK to be less accurate and precise than those for wildfowl. However, it is suggested that some hundreds of thousands of terrestrial game birds may die from lead poisoning annually. These estimated numbers are mainly due to the large numbers of pheasants and other game birds bred and released to be killed for sport in the UK every year (about 50 million—DEFRA 2013; Larkman et al. 2015). Even if the proportion of birds with ingested shot at a given time is only a few percent, several million birds are likely to ingest gunshot in the course of a year in the UK. It is possible that some birds (e.g. pheasants) may be less sensitive to the effects of lead than other terrestrial birds or waterbirds (as described in previous sections), and this may affect estimates of the numbers dying because of lead poisoning.

Most birds that ingest lead from ammunition probably suffer some effect on their welfare. These effects are likely to be severe in birds that subsequently die of lead poisoning. As described earlier, some of the lead that is embedded in the flesh of birds that have been shot but survive may also be absorbed into the blood and result in elevated PbB, albeit to a far lesser degree than ingested lead gunshot. Should this be the case, numbers of birds suffering welfare effects may be higher than current estimates. In the EU an estimated 6 million waterfowl are shot annually (AMEC 2012). While crippling ratios (the number of birds injured to those killed) are variable (e.g. Clausen et al. 2017), numbers crippled and carrying embedded gunshot may be large and may number over a million waterfowl, along with many more terrestrial birds. However, there is currently insufficient evidence to evaluate either the severity of impacts or numbers of birds potentially affected by embedded gunshot with any certainty.

Insufficient information exists to estimate numbers of raptors impacted in Europe and elsewhere although many scavenging and predatory species are affected (Table 2; Mateo 2009; Golden et al. 2016), probably including some currently unstudied species. This is consistent with what would be predicted from the source of lead and pathway of exposure.

### Effects on populations

In some countries, there has been considerable debate about effects of lead from ammunition on bird population size and trend. Absence of robust information on this topic is sometimes cited as a reason for political inaction (Truss 2016). Any additional mortality of wildlife has the potential to affect population size and trends to some extent, and is certain to do so in the absence of complete, or perfect, density dependence. If density dependence exists but is not complete, population size will be lower because of lead poisoning, but it will stabilise at this lower level if the strength of density dependence is sufficient. If density dependence is absent or weak, any additional mortality, including that caused by lead poisoning, will cause a previously stable population to decline markedly or go extinct. Only if there is perfect density dependence which operates on demographic rates within the annual cycle and after the time when lead poisoning occurs, would deaths caused by lead poisoning be completely compensated for by density-dependent enhancement of survival or breeding success. Only then would lead poisoning be expected to have no effect on population size. It is difficult to measure the strength and form of density dependence in wild populations, so it is rarely known with any precision how large the effect of additional mortality on population size will be. However, a given proportion of birds poisoned is likely to have the biggest impact on species that naturally have the lowest annual mortality and reproductive rates, such as eagles and vultures.

Perfect or complete density dependence has rarely been demonstrated and is thought to be rare in the bird species affected by lead poisoning. For example, exhaustive studies of the effects of hunting on populations of mallards in North America provide no indication that the additional mortality it causes are compensated for by density-dependent processes (Nichols et al. 2015). Therefore, it is reasonable and precautionary to take evidence of additional mortality as evidence of an effect on population size and trend. Cases of populations exposed to ammunition-derived lead in which density dependence is clearly too weak to compensate for deaths caused by lead poisoning include those of the California condor in California and in the Grand Canyon. Both of these populations would decline to extinction because of poisoning from ammunition-derived dietary lead were it not for the capture, diagnosis and veterinary treatment of affected animals (Green et al. 2008).

**Fig. 3:**
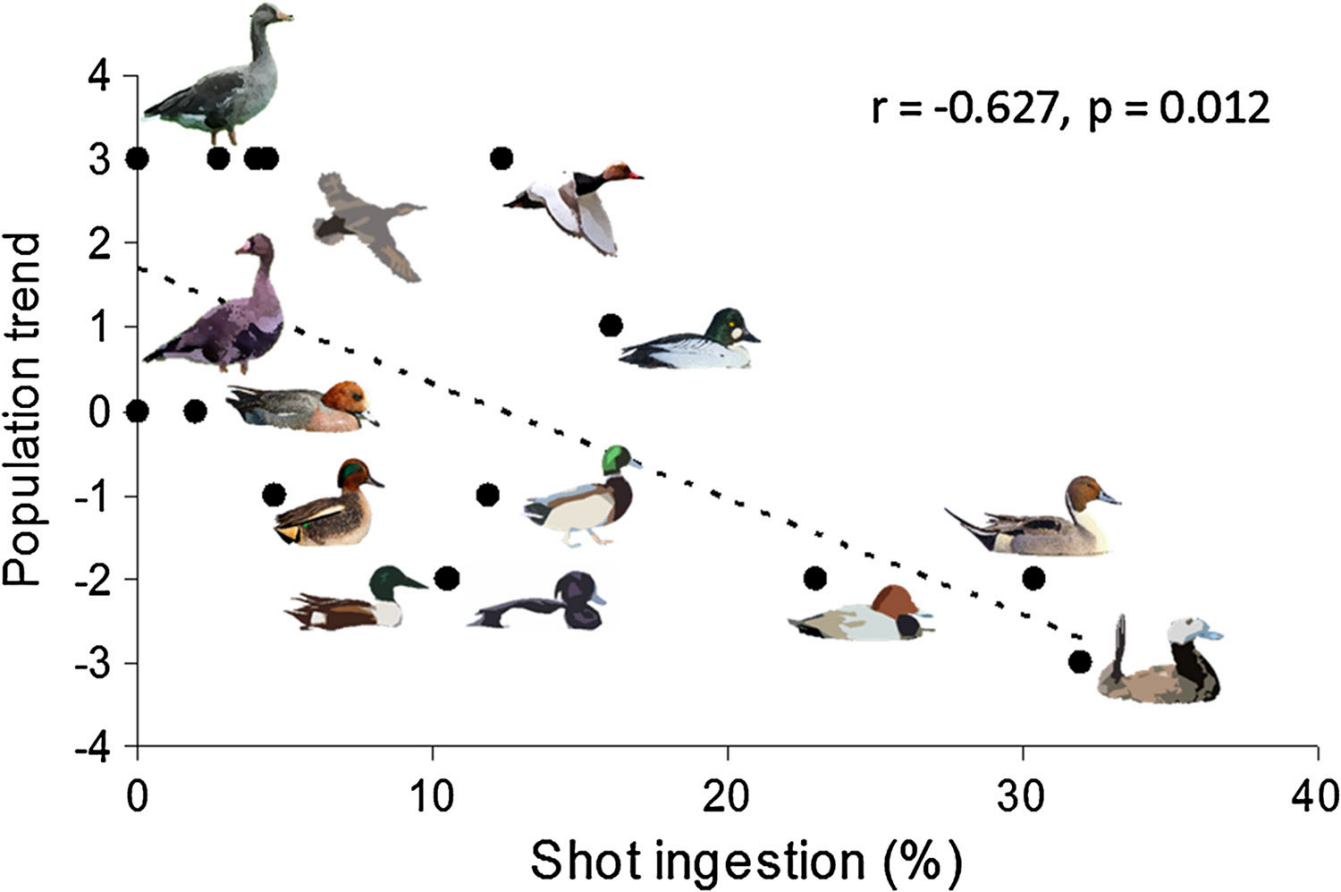
Correlation between the prevalence of lead shot ingestion and the trend of the wintering population in Europe of 15 species of waterfowl. Modified from Mateo (2009)

Another approach to the assessment of population-level effects of lead poisoning is to correlate variation in population growth rates observed among geographical regions or species with levels of exposure to lead. Green and Pain (2016) found that for the eight duck species that winter in freshwater habitats in the UK, inter-specific variation in mean annual population growth rate during the period 1990/1991 to 2013/2014 was significantly negatively correlated with two independent measures of the prevalence of ingested lead gunshot in the UK and Europe. This relationship was also found for annual growth rates in the period 1966/1967 to 2013/2014, derived from smoothed population trajectories and was insensitive to the choice of period over which the effect was investigated. Duck species with high prevalence of ingested lead were more likely to have undergone a long-term population decline. These findings support the hypothesis that ingested lead gunshot affects population trend. The authors expressed particular concern about the possible impact of ingested lead gunshot on the common pochard, a species now listed as globally threatened (Vulnerable) because of population declines (BirdLife International 2015). Previously, the prevalence of lead shot ingestion was found to be negatively correlated with population trends in 15 waterbird species across Europe, in the last decades of the twentieth century (Fig. 3).

Conducting a modelling study of spectacled eider (*Somateria fischeri*), Flint et al. (2016) suggested that populations would respond most dramatically to changes in adult female survival and that reductions in adult female survival related to lead poisoning were locally important. As most mortality from lead exposure occurs over winter, the related reduction in adult survival may be impeding recovery of local populations.

Meyer et al. (2016) used population models to create example scenarios demonstrating how mortality from lead poisoning and other poisons might affect the populations of three susceptible species: grey partridge (*Perdix perdix*) in continental Europe, common buzzard in Germany and red kite in Wales. Lead gunshot ingestion and poisoning at modelled levels (4–16% for lead poisoning depending on species) affected populations by reducing population size and slowing population growth. Lead gunshot alone reduced the population size of grey partridges by 10%, and reduced annual growth rate of the red kite population from 6.5% to 4%, slowing recovery. Decrease in the common buzzard mean population size by lead gunshot and poisons combined was much smaller (≤ 1%). The effects are somewhat higher if ingestion of these substances additionally causes sub-lethal reproductive impairment (which we know that lead can do—as described above). While these results are subject to uncertainty, they suggest that declining or recovering populations are most sensitive to poisoning by lead from ammunition or other poisons. These example scenarios may not be replicable to other places where exposure levels differ but they do illustrate how poisoning can hypothetically affect population levels and growth rates.

Population-level effects of lead poisoning are also supported by previous research on lead fishing weights that were responsible for widespread lead poisoning in mute swans. Following a ban on the sale and use of most lead fishing weights in England and Wales in 1987 there was a sharp reduction in most areas in the numbers of mute swans dying or sick from lead poisoning. This has been considered crucial to the subsequent increase in the species’ population (Sears and Hunt 1991; Kirby et al. 1994; Perrins et al. 2003). In a long-term study (1982–2012) lead fishing weights have recently been shown to have a population-level effect on common loons (*Gavia immer*) in New Hampshire, USA where their ingestion was responsible for 48.6% of adult loon deaths. The authors modelled the loon population retrospectively and estimated that mortality caused by ingestion of lead tackle reduced the population growth rate by 1.4% and the state-wide population by 43% during the years of the study (Grade et al. 2018).

The known effects of lead upon physiology, behaviour and reproduction, the widespread mortality in birds known to occur as a result of exposure to lead from ammunition, and the recent studies described here provide compelling evidence that lead from ammunition can, and sometimes does, negatively affect population levels and trends, and not only in quarry species. For this reason, it is reasonable and precautionary to take evidence of additional mortality as a result of lead poisoning as evidence of population-level effects.

## Conclusions

Recent research supports and supplements more than a century of work on lead poisoning from ammunition sources in birds. The results of this work show that lead poisoning of birds is likely to occur wherever lead ammunition is used and a pathway of exposure exists. Cases of lead poisoning in new species and countries simply reflect that these had not previously been studied. Taxa beyond birds are also affected; recent research highlights risks to mammalian scavengers and predators although this has not been extensively investigated. Trends in lead poisoning research in birds reflect those in human medicine, with effects detected at ever lower levels of lead exposure and absorption. Large numbers of wild birds suffer welfare effects and are killed by spent lead ammunition annually, and it affects populations. Alternative non-toxic ammunition exists and some has been in widespread use for decades. Removing this avoidable source of environmental contamination and suffering and mortality of wildlife is a matter of political will (e.g. Arnemo et al. 2016; Kanstrup et al. 2018).
